# Phytochemical Analysis and *In-vitro* Bioactivity of *Scrophularia umbrosa * Rhizome (*Scrophulariaceae*)

**Published:** 2018

**Authors:** Elhameh Nikkhah, Fariba Heshmati Afshar, Hossein Babaei, Parina Asgharian, Abbas Delazar

**Affiliations:** a *Drug Applied Research Center, Tabriz University of Medical Sciences, Tabriz, Iran. *; b *Faculty of Pharmacy, Tabriz University of Medical Sciences, Tabriz, Iran. *; c *Student Research Committee, Faculty of Pharmacy, Tabriz University of Medical Sciences, Tabriz, Iran.*

**Keywords:** Antimalaria, Antioxidant, TPC, TFC, GC-MS, Scrophularia umbrosa

## Abstract

*Scrophularia umbrosa* is a medicinal plant used as a traditional herb. This study was designed to investigate the phytochemical analysis of methanol (MeOH), DCM, and n-Hexane extracts of rhizome as well as total phenol and total flavonoid contents (TPC and TFC). *In-vitro* β-hematin formation assay and DPPH method were applied for analyzing antimalarial and free-radical scavenging activities of the extracts, respectively. The formation of hemozoin has been proposed as an ideal drug target for antimalarial screening programs. The results showed that n-hexane and MeOH extracts of rhizome had no significant inhibitory effect on heme biocrystallization whereas the DCM extract of rhizome showed moderate antimalarial activity in comparison with chloroquine. GC-MS data showed that volatile portions of DCM and n-Hexane extracts from *Scrophularia umbrosa (S. umbrosa) *contained a few identifiable compounds. Moreover, fractions 20% and 40% MeOH-Water of MeOH extract of *S. umbrosa* displayed moderate to strong free radical scavenging activity which showed a positive relation between phenolic and flavonoid contents and free radical scavenging activity. Based on the results, the fractions of MeOH extract were evaluated by ^1^HNMR for predicting the groups of natural compounds and interfacing of chemical and biological assessments.

## Introduction

In recent decades, the plant kingdom has been considered as the important source of potential drugs which are easily available, safe, and inexpensive and rarely show side effects (1). Based on estimations of the World Health Organization, more than 80 percent of people still rely mostly on traditional drugs such as plants for treating their aliments (2). So, nowadays, several studies have been carried out to provide scientific support for the effectiveness of herbal medicines. *Scrophularia* genus belongs to *Scrophulariaceae* family including about 3000 species and 220 genera (3-5). *Scrophularia L*. consists of about 200 species of herbaceous flowering plants, commonly known as ‘figwort’. It occurs throughout mountainous regions, forests riversides (*e.g.* *S. umbrosa*) and rarely in hot deserts (6). *Scrophularia umbrosa Boiss* with common name “water figwort” is one of the species which is native to Iran and many species of this genus are widely distributed in various regions of Iran (6, 7). *S. umbrosa* possesses quadrangular and winged stems which are characteristics of figwort genus. Many *Scrophularia *plants have been used in Asian countries as a medicinal herb for the treatment of various diseases such as allergy, cancer, rheumatics, cardiovascular and chronic inflammatory disorders (8-10). These species also have been found to have numerous biological activities such as antibacterial, antiprotozoal, antitumor, hepatoprotective, and diuretic properties, and have been used in the treatment of mental, nervous and gastrointestinal disorders (11-14). Furthermore, some species of this genus have been used to treat eczema, wounds, goiter, ulcers, cancer, and fistulae (15). Several species of this genus evaluated phytochemically, showing the presence of biologically active phenylethanoids, phenylpropanoids, flavonoids, iridoid glycosides, and terpenoids (16-19). Based on previous reports and as part of our on-going studies on Iranian medicinal plants, we have studied the MeOH, DCM, and n-Hexane extracts from rhizome of *S. umbrosa*. To the best of our knowledge, no research has been conducted on pharmacological and biological activities or chemical composition of this plant. In this paper, we report the antimalarial and free radical scavenging activities of MeOH, DCM and n-Hexane extract from rhizome of this plant, as well as total phenol and total flavonoid contents. Besides, GC-MS and NMR techniques were used for the identification of the components of the extracts.

## Experimental

*Chemicals*

Folin ciocaltea reagent and gallic acid were purchased from Fluka. DPPH was obtained from Sigma, Germany. All other solvents and chemicals were analytical and HPLC grade.

*Plant Material*

The rhizomes of *S. umbrosa* were collected from Mishodaghi mountain at E: 45° 47’ 24”, N: 38° 20ʹ 59” (altitude of 1780) in East Azarbaijan province during the flowering period. Botanical identification with voucher No: (Tbz-Fph-762) was carried out at the Herbarium of Faculty of Pharmacy, Tabriz University of Medical Sciences, Tabriz, Iran.

*Extraction and Isolation*

The dried and ground rhizomes of *S. umbrosa* (100 g) were extracted with a Soxhlet apparatus with n-hexane, DCM and MeOH, successively. Obtained extracts were separately concentrated by rotary evaporator at a maxium temperature of 45 ºC yielding 0.34 g, 3.43 g, and 11.70 g from each extract, respectively. 

A portion of the dried MeOH extract (2 g) was subjected to solid-phase extraction (SPE) on Sep-Pak (Vac 35 mL; , 10 g; C_18_ cartridges, Waters, Ireland) using a step gradient of MeOH: Water mixtures (10:90, 20:80, 40:60, 60:40, 80:20 and 100:0) as eluent. Solvent of fractions were separately removed using a rotary evaporator at a maximum temperature of 45 °C. In order to purify and isolate phytochemials, the SPE fraction eluted with 10% MeOH was analyzed by reversed-phase preparative HPLC (Knauer, preparative pump 1800, with photodiode array detector PDA, equipped with a Reprosil 100 C18,250 mm length, 20 mm i.d, particle size 10 μm, Dr. Maisch column, Germany) using the mobile phase: 0-50 min, linear gradient of 10-30% MeOH in water; 0-40 min, maintained at 10% MeOH in water, to isolate compound [1] (82.8 mg**, ***t*_R _= 21.5 min)**, **[2] (0.8 mg**, ***t*_R _= 37.5 min). In all above prep-HPLC analyses, the flow rate of the mobile phase was 8.0 mL/min. The structures of all compounds were elucidated unequivocally by spectroscopic means and comparing with references. It afforded two iridoid structures, which were identified unequivocally as aucubin (1) and lamalbide (2).

Aucubin [1]: amorphous powder, ^1^H- NMR (400 MHz, D_2_O): δ 5.16 (d, 1H, J = 5.02 Hz, H-1), 6.1 (dd, 1H, J = 6.10 Hz, H-3), 5 (dd, 1H, J = 3.56, 2.56 Hz, H-4), 3.03 (t, 1H, J = 5.80 Hz, H-5), 4.43 (brs, 1H, H-6), 5.74 (brs, 1H, H-7), 2.67 (m, 1H, H-9), 4.20 (dd, 2H, J = 15.22, 16.68 Hz, H-10), 4.46 (1H, H-1ʹ), 3.60 (dd, 1H, J = 5.50, 6.80 Hz, H-6ʹa), 3.80 (dd, 1H, J = 1.40, 10.83 Hz, H-6ʹb), 3.20-3.40 ( remaining sugar protons). ^13^CNMR (400 MHz, D2O): 94.19(C-1), 138.39(C-3), 104.08(C-4), 41.16(C-5), 79.37(C-6), 127.34(C-7), 145.69(C-8), 45.19(C-9), 58.33(C-10), 97.23(C-1ʹ), 71.61(C-2ʹ), 74.51(C-3ʹ), 68.41(C-4ʹ), 75.04(C-5ʹ), 59.5(C-6ʹ). Data were in agreement with the published reports (20).

Lamalbide [2]: amorphous powder, ^1^H- NMR (400 MHz, D2O): δ 5.49 (brs, 1H, H-1), 7.30 (s, 1H, H-3), 2.77 (dd, 1H, J = 10.80, 3.20Hz, H-5), 3.9 (t, 1H, J = 4.02 Hz, H-6), 3.5 (d, 1H, J = 4.45 Hz, H-7), 2.67 (d, 1H, J = 11.21 Hz, H-9), 1.05 (s, 3H, H-10), 3.59 (s, 3H, H-OCH_3_), 4.61 (d, 1H, J = 8.06 Hz, H-1ʹ), 3.77 (dd, 1H, H-6ʹa), 3.56 (dd, 1H, H-6ʹb), 3.00-3.34 (remaining sugar protons). Data were in agreement with the published papers (21, 22).

*Free radical scavenging activity*

The ability of the extracts and fractions to scavenge radicals was assessed by the method is based on the reduction of DPPH (molecular formula C_18_H_12_N_5_O_6_) solutions in the presence of a hydrogen donating antioxidant. DPPH (8 mg) was dissolved in appropriate solvent (methanol or chloroform) to obtain a concentration of 80 μg/mL. For preparing the test samples, the MeOH extract and SPE fractions were dissolved in methanol to obtain a concentration of 1 mg/mL whereas. The DCM and n-Hexane extracts were dissolved in chloroform. Dilutions were made to obtain different concentrations of extracts and fractions and then diluted solutions (5 mL each) were mixed with DPPH (5 mL). After a 30 min incubation period at room temperature, the absorbance was read against a blank at 517 nm with a Shimadzu UV/Visible Spectrophotometer 160A (USA). The percentage reduction was plotted against the sample extract concentration in order to calculate RC50 values which is the extract concentration providing 50% loss of DPPH activity. Quercetin was used as positive control and all tests were carried out in duplicate (23-25).

*Total Phenol Content (TPC)*

Total phenolic contents of DCM, n-Hexane and MeOH extracts as well as MeOH fractions were determined by the modified Folin- Ciocalteau assay as gallic acid equivalents (GAE) (26). One mL of extracts samples (5 mg in acetone: water (60:40) v/v) were mixed with 0.2 mL Folin- Ciocalteau`s reagent (1:2 diluted with water) and 1 mL of 2% Na_2_CO_3_ was added to the mixture. As control, reagent without adding extract was used. After incubation of the samples at room temperature for 30 min, their absorbance was measured at 750 nm (Pharmacia biotech Ultrospec 2000, UV/Visible spectrophotometer, England). For the calibration curve, 10 mg of Gallic acid was dissolved in 10 mL of acetone: water (60:40) v/v as a stock solution. Different dilutions of gallic acid were prepared and then determined by Folin- Ciocalteau`s method. Experiments were repeated 2 times for every dilution and a calibration curve was drawn.

*Total Flavonoid Content (TFC)*

The flavonoid content of DCM, n-Hexane and MeOH extracts as well as MeOH fractions were determined using a modified colorimetric assay (27) and used rutin as a standard. Extracts or standard solutions (0.5 mL) were mixed with distillated water (2 mL) and 5% NaNO_2_ (150 μL). After six min, test mixtures were combined with 10% AlCl_3_ solution (150 μL), 4% NaOH (2 mL) and finally distillated water was added to adjust the volume of 5 mL. The absorbance of the samples was read at 510 nm against blank after 30 min at room temperature and the total flavonoid content was expressed as rutinoside equivalents in mg per 100 mg of dried extract.

*In-vitro β-hematin formation assay (antimalarial test)*

The potential antimalarial activity of plant extracts was evaluated by the method described by Afshar *et al.* (28) with some modifications. Briefly, varying concentrations of the extracts were incubated with 300 μM of hematin (freshly dissolved in NaOH), 10 mM oleic acid and 10 μM HCl. The reaction volume was adjusted to 1 mL using 500 mM sodium acetate buffer, pH 5. Chloroquine diphosphate was used as a positive control. The samples were incubated overnight at 37 °C with regular shaking. After incubation, the samples were centrifuged (14,000 × g, 10 min, at 21 °C) and the hemozoin pellet repeatedly washed with sonication (30 min, at 21 °C; FS100 bath solicitor; Decon Ultrasonics Ltd.) in 2.5% (w/v) SDS in phosphate buffered saline followed by a final wash in 0.1 M sodium bicarbonate, pH 9.0, until the supernatant was clear (usually 3-5 washes). After the final wash, the supernatant was removed and the pellets were re-suspended in 1 mL of 0.1 M NaOH before determining the hemozoin content by measuring the absorbance at 400 nm (Beckmann DU640 spectrophotometer). The results were recorded as % inhibition (I%) of heme polymerization/crystallization compared to positive control (chloroquine) using the following formula: 

I% = [(AN–AA)/AN] × 100

AN: absorbance of negative control;

AA: absorbance of test samples.

*GC-MS and GC-FID analyses*

DCM and n-Hexane extracts of rhizome from *S. umbrosa* were analyzed using a Shimadzu GCMS-QP5050A gas chromatograph-mass spectrometer (GC-MS) fitted with a fused methyl silicon DB-1 column (60 m × 0.25 mm id, 0.25 μm film thickness). Helium was used as carrier gas at a flow rate of 1.3 mL/min. The column temperature was kept three min at 50 °C, increased to 260 °C at a rate of 3 °C/min, and finally kept 5 min at 260 °C. The injector temperature was 240 °C and the split ratio was adjusted at 1:19. The injection volume was 1 μL (10 mg/mL in n-hexane) for analysis. The mass spectral (MS) data were obtained at the following conditions: ionization potential 70 eV; ion source temperature 200 °C; quadrupole temperature 100 °C; solvent delay, 2 min; resolution 2000 amu/s and scan range 30-600 amu; EM voltage 3000 volts. Identification of compounds was based on direct comparison of the Kovats Indices (KI) and MS data with those for standard alkanes, and computer matching with the NIST NBS54K library, as well as by comparison with references (29, 30). For quantitation (area%), the GC analysis was also performed on a Shimadzu GCMS-QP5050A gas chromatograph fitted with a FID detector. 

*Statistical Analysis*

All experiments were conducted in duplicate and triplicate measurements and represented as the mean ± standard deviations. Data were analyzed by Excel 2007 Microsoft. The RC_50_ values were calculated from linear regression analysis. 

## Results and Discussion

**Figure 1:**
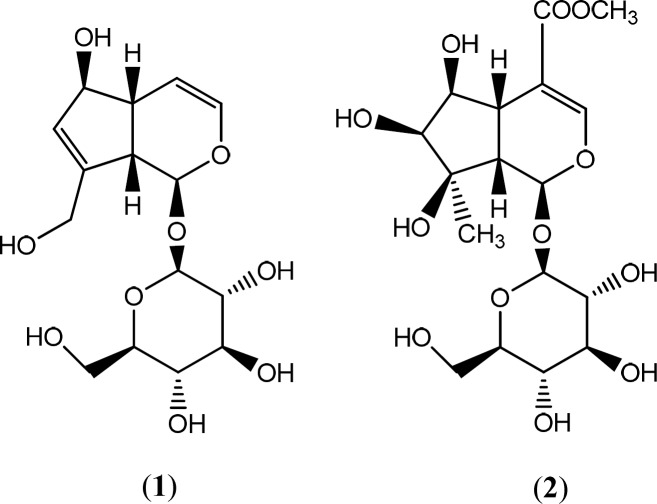
Sturacture of iridoid compounds, (1) aucubin and (2) lamalbide isolated from *S. umbrosa*

A combination of solid phase extraction (SPE) and reversed-phase prep-HPLC analyses of the MeOH extract obtained from the rhizomes of *S. umbrosa* led to the characterization of two known iridoid glycosides aucubin [1] (20) and lamalbide [2] (Figure 1) (21, 22). The chemical structures of all isolated compounds were elucidated unequivocally through ^1^HNMR and ^13^CNMR. All spectroscopic data were in agreement with respective published data.

The results of free radical scavenging activity, total phenolic and total flavonoid contents obtained from extracts and MeOH fractions are given in Table 1.

Free radical scavenging activity of the n-Hexane, DCM and MeOH extracts and respective fractions were determined by DPPH method. In this assay, the extracts were able to reduce the stable radical DPPH to the yellow colored diphenylpicryl hydrazine. The method is based on the reduction of methanolic DPPH solution in the presence of a hydrogen-donating scavenger through the formation of the non-radical form DPPD-H (31). It was found that all extracts and fractions reduced DPPH radicals in a concentration-dependent manner. The lower RC_50_ values indicates a stronger ability of the antioxidant substance to scavenge the DPPH radicals while the higher RC_50_ values indicates a lower scavenging activity of the scavengers. Compared to the standard antioxidant Quercetine (RC_50_ 0.004 ± 0.500 mg/mL), n-Hexane, DCM and MeOH extracts as well as related fractions exhibited weak to strong radical scavenging activities (RC_50_ 0.019-1.904 mg/mL), but the highest free radical scavenging activity belonged to 20% MeOH-water fraction of rhizome (RC_50_ 0.019 ± 0.0004).

The radical scavenging activity in the rhizome extract and respective fractions decreased in the following order: 20% MeOH-water > 40% MeOH-water > MeOH > 60% MeOH-water > 80% MeOH-water > DCM > 100% MeOH > n-Hexane > 10% MeOH-water.

Polar extracts (MeOH) showed stronger activity than non-polar extracts. The MeOH extracts might possess phenolic compounds like, flavonoids, coumarins or phenyl propanoids, which might have contributed toward the significant free radical scavenging activity of these extracts.

Total phenolic content was determined in comparison with standard Gallic acid and the results expressed in terms of mg GAE/100 g dry sample in Table 1. According to the results, it was found that 40%, 20% MeOH-water fractions and MeOH extract (29.27 ± 3.663, 22.20 ± 1.980 and 14.57 ± 2.574 mg GAE/100 mg, respectively) contained more phenolic contents than the others. Other fractions generally possessed low total phenolic contents with the range of 4.35 ± 0.000 to 8.06 ± 2.871 mg GAE/100 mg of extract powder. 

**Table 1:** Total phenol content (TPC), total flavonoid content (TFC) and antioxidant activity of the DCM, n-Hexane and MeOH extract and respective fractions of *S. umbrosa*

**Extracts and Fractions**	**Total Phenol Content (TPC) (mg/100 mg)**	**Total Flavonoid Content (TFC) (mg/100 mg)**	**Free radical scavenging Activity (RC**_50_ **) (mg/L)**
n-Hexane	4.35 ± 0.000	3.62 ± 0.257	1.44 ± 0.224
DCM	8.06 ± 2.871	2.21 ± 0.008	0.35 ± 0.008
MeOH	14.57 ± 2.574	5.02 ± 0.577	0.06 ± 0.001
10% MeOH-water	6.10 ± 0.792	0.74 ± 0.016	1.90 ± 0.035
20% MeOH-water	22.20 ± 1.980	10.33 ± 0.062	0.02 ± 0.0004
40% MeOH-water	29.27 ± 3.663	8.39 ± 1.052	0.02 ± 0.019
60% MeOH-water	7.50 ± 3.069	1.46 ± 0.016	0.25 ± 0.012
80% MeOH-water	5.61 ± 0.099	4.21 ± 0.210	0.27 ± 0.012
100% MeOH-water	4.84 ± 0.396	2.40 ± 0.047	0.36 ± 0.228
Quercetine	-----	-----	0.005 ± 0.0009
Rutin	-----	-----	0.00975 ± 7.07E-05

**Table 2:** The 50% and 90% inhibition concentration (mg/mL) of DCM extracts of *Scrophularia umbros*a in β-hematin formation assay

**Extract**	**Yields (%)**	**IC**_50_ ** (mg/mL)**	**IC**_90_ ** (mg/mL)**
DCM extract of Rhizome	3.43	2.71 ± 0.88	3.586 ± 1.41
Chloroquine	-	0.013 ± 0.003	0.163 ± 0.004

**Table 3:** Chemical profile of the DCM and n-Hexane extract of rhizome from *S. umbrosa*

**No.**	**Compounds**	**K. I.**		**Real Area%**	
DCM extract					
1	cinnamic acid	1113		27.07	
2	3-(4-methoxyphenyl)-2-Propenoic acid	1614		8.53	
3	Hexadecanoic acid (Palmitic acid)	1915		8.78	
4	9,12-Octadecadienoic acid	2106		44.61	
Total Identified				88.99	
**n-Hexane extract**					
1	Caprylic acid		1106		0.62
2	(E,E)-2,4-Decadienal		1209		3.46
3	Hexadecanoic acid (Palmitic acid)		1916		24.69
5	(E,E)-9,12-Octadecadienoic acid, methyl ester		2077		18.61
6	(Z,Z,Z )-8,11,14-Eicosatrienoic acid,		-		16.55
7	2-Methyl-Z,Z-3,13-octadecadienol		-		4.90
8	Heneicosane		2100		2.21
9	γ-Sitosterol		3220		15.45
Total identified					86.49

**Table 4:** Prediction of main chemical groups of natural compound in the MeOH extracts of *S. umbrosa* and their fractions based on ^1^HNMR spectra

**Extract and Fractions**	**Predicted Compounds**
MeOH Extract	Mixture of phenols , sugars, *etc*
10% MeOH-water fraction	Iridoids
20% MeOH-water fraction	phenylethanoids
40% MeOH-water fraction	Flavonoids
60% MeOH-water fraction	phenylethanoids
80% MeOH-water fraction	Not defined
100% MeOH fraction	Not defined

**Figure 2:**
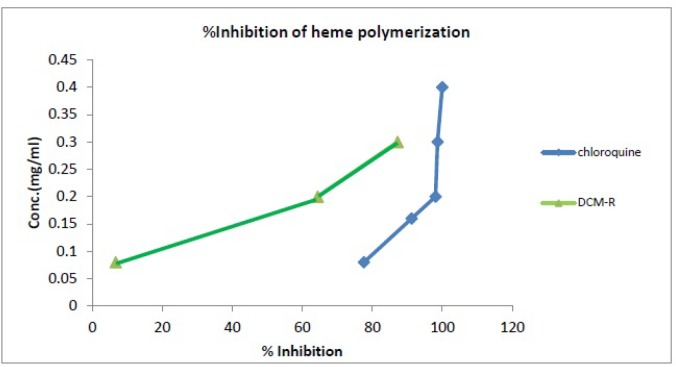
Comparison of % inhibition of heme polymerization between DCM extract and chloroquine solution.

Total flavonoid content (TFC) was determined in comparison with standard rutinoside and the results expressed in terms of mg rutinoside per mg dry powder. The TFC values for 20% and 40% MeOH-water fractions were 10.33 ± 0.062 and 8.39 ± 1.052 mg per 100 mg dry sample, respectively. Total flavonoid contents of other extracts and fractions are shown in Table 1. 

Based on several researches, there is a positive relation between total phenol contents and free radical scavenging activity of the plants material (32-34). Flavonoids with numerous structures including flavonols, flavones, and condensed tannins, are a class of plant phenolics, which contain hydroxyl groups. They are responsible for the free radical scavenging and chelating properties (35, 36). According to our findings, total flavonoids and total phenol of 20%, 40% and 60% MeOH-water fractions was higher than the other fractions in parallel to the free radical scavenging activity of these fractions. It has been demonstrated that the flavonoids with polyhydroxylated substitution on rings A and B, showed their free radical scavenging activities by donating hydrogen atoms and thereby scavenging the free radicals generated during lipid peroxidation (36, 37). Different kinds of phenolic compounds display different radical scavenging capacities which are directly related to their chemical structure. As an illustration, the previous researches showed that the phenolic compounds with ortho- and para- di hydroxylation or a hydroxyl and a methoxy group or both have more potent radical scavenging activity than simple phenolic (33). Moreover, the presence of double bond conjugated and ketone groups in the whole molecule might play different polarities in the structure of the antioxidants and can be attributed to their fee radical scavenging activity (38). The sensitivity of folin ciocalteu reagent to a broad range of phenolic compounds might be the other factor that lead to this results because this reagent react both free phenolic and bound phenolic in extracts and other samples, but the DPPH free radicals show different sensitivity to various radical scavengers; therefore, the DPPH assay just determined free antioxidants and phenolic (39). It seems that further studies are needed for the isolation and elucidation of the structure of phenolic components and also more investigations are necessary for better understanding of their mechanism of action as antioxidants and radical scavengers.

In this study, total extracts of rhizome were assessed for their antimalarial activity using the *in-vitro* β-hematin formation assay. The n-hexane and MeOH extracts showed no significant inhibitory effects on heme biocrystallization properties; however, the DCM extract showed moderate antimalarial effect in comparison with the negative control (Table 2 and Figure 2). At higher concentrations (> 3 mg/mL), the DCM extracts strongly inhibited heme bio crystallization and illustrated potent antimalarial effects. The IC_50_ value of the DCM extracts was 27.12 (rhizome) mg/mL and that of the positive control chloroquine was 0.014 mg/mL. Malaria-infected erythrocytes are characterized by a high rate of production of ferri proto porphyrin IX (heme) as by product of the ingestion and digestion of host cell hemoglobin (40). Hemoglobin is used as parasite primary nutrition source during intra erythrocyte development and proliferation (41); however, heme is generated as a toxic material of this process (42). Consequently, cellular metabolism is affected by inhibiting enzymes, per oxidizing membranes, and producing oxidative free radicals (43). Hence, malaria parasite detoxifies heme in its food vacuoles by converting it to the dark micro crystals of hemozoin which commonly referred to as malaria pigment (43-45). Earlier this process used to be known as “heme polymerization” (42); however, it has been revealed that the structure of hemozoin (and its synthetic equivalent, β-hematin) is a cyclic dimer of ferriprotoporphyrin IX (heme). Several studies have shown that chloroquine and most of other antimalarial compounds inhibit β-hematin formation under different conditions (45). The heme biocrystallization and inhibition assay is based on the above facts (46, 47).

The chemical composition of the DCM and n-Hexane extract of rhizome was determined by the GC-MS analysis and identified based on direct comparison of KI and MS data with those for standard compounds, and computer matching with the NIST, NBS54K Library, as well as by comparison with references (11, 29 and 30) (Table 3). 

GC-MS data showed that volatile portions of DCM and n-Hexane extracts contained a few identifiable compounds. High proportion of the volatile part of DCM extract (44.61%) consisted of Linoleic acid. Cinnamic acid, a phenolic compound, is the other main component which was present remarkably in DCM extract (27.07%). Based on previous studies, the formation of hemozoin in the parasite food vacuole is initiated by lipid compounds which can induce hemozoin biocrystalization. An oxidative mechanism has been demonstrated for lipid-mediated β-hematin formation, which might be mediated by production of some free radical intermediates of heme (48). In another study, a commercially available lipid, lecithin, a source of phospholipids containing about 50% unsaturated fatty acids, possessed remarkable effects as an initiator for β-hematin formation (49); therefore, the possibility that the antimalarial activity displayed by DCM extract observed here would be due to the presence of 9, 12-Octadecadienoic acid (linoleic acids) and Hexadecanoic acid (Palmitic acid) that could not be excluded.

As can be seen in Table 4*, *the results of ^1^HNMR spectroscopy are parallel with findings that have been reported in Table 1. Fractions which show peaks in aromatic regions at δ_H_ 6-8 ppm, possess polyphenolic compounds and lower RC_50_ values. The ^1^HNMR spectera of 10% MeOH-water fractions belonging to *S. umbrosa *have revealed that there is no flavonoid compounds in this fraction but some other phenols may exist in low concentration in this fraction that cause antioxidant activity. Obviously the spectrums showed that flavonoid compounds exist in high concentrations in 40% and 60% MeOH-water fractions of this plant. For this reason further studies are needed for the isolation and elucidation of the structure of phenolic components and also more investigations are necessary for better understanding of their mechanism of action as radical scavengers.

Solid phase extraction of the polar extract from *S. umbrosa* followed by reversed-phase preparative HPLC analysis of the 10% fraction of MeOH extract led to identification of iridoid glycoside structures. Compounds 1 and 2 were identified as aucubin (1) and lamalbide (2) by direct comparison of their spectroscopic data with those published in the literature (21, 22, 50-53).

## Conclusion

The results presented above demonstrated that some fractions (20%, 40% and 60%) of MeOH extracts of *S. umbrosa* had moderate to strong antioxidant activity and it is possible to conclude that there is a positive relation between phenolic and flavonoid composition and antioxidant activity. Additionally, based on the results of the current study, the conclusion can be drawn that fractionation of extracts and running their ^1^HNMR could be valuable method for predictiion of natural compounds and interfacing of chemical and biological assessments. By the way, investigations are in process to identification of the structure of these phenolic and flavonoids.
